# HOW THE SAME GENETIC PROGRAM RUNS DIFFERENTLY IN INDIVIDUAL ANIMALS TO AFFECT AGING AND DISEASE

**DOI:** 10.1093/geroni/igac059.1067

**Published:** 2022-12-20

**Authors:** Alexander Mendenhall, Bryan Sands, Soo Yun

**Affiliations:** University of Washington, Seattle, Washington, United States; University of Washington, Seattle, Washington, United States; University of Washington, Seattle, Washington, United States

## Abstract

Monozygotic human twins will age at different rates. The same is true for isogenic laboratory animals. Some of these differences in the rates of aging are caused by differences in the expression of genes. And, some of the differences in gene expression between isogenic individuals are caused by seemingly non-heritable, stochastic epigenetic differences. Here we discuss how differences in chaperone expression can influence aging and a model of Ras-driven neoplasia risk and survival in the model nematode Caenorhabditis elegans. We review evidence suggesting differences in epigenetic silencing machinery contribute to differences in chaperone gene expression. We suggest models for germline and somatic epigenetic regulation of chaperones. We discuss potential means of targeted epigenome modification, and potential implications for human health during aging.

